# Comparative Genomic and Phenotypic Analysis of the Vaginal Probiotic *Lactobacillus rhamnosus* GR-1

**DOI:** 10.3389/fmicb.2018.01278

**Published:** 2018-06-15

**Authors:** Mariya I. Petrova, Jean M. Macklaim, Sander Wuyts, Tine Verhoeven, Jos Vanderleyden, Gregory B. Gloor, Sarah Lebeer, Gregor Reid

**Affiliations:** ^1^Centre of Microbial and Plant Genetics, KU Leuven, Leuven, Belgium; ^2^Department of Bioscience Engineering, University of Antwerp, Antwerp, Belgium; ^3^Canadian Research and Development Centre for Human Microbiome and Probiotics, Lawson Health Research Institute, The University of Western Ontario, London, ON, Canada; ^4^Department of Biochemistry, The University of Western Ontario, London, ON, Canada; ^5^Department of Microbiology and Immunology, The University of Western Ontario, London, ON, Canada; ^6^Department of Surgery, The University of Western Ontario, London, ON, Canada

**Keywords:** adaptation, genome, lactobacilli, probiotics, vaginal niche

## Abstract

*Lactobacillus* represents a versatile bacterial genus, which can adapt to a wide variety of ecological niches, including human body sites such as the intestinal and urogenital tract. In this study, the complete genome sequence of the vaginal probiotic *Lactobacillus rhamnosus* GR-1 was determined and compared to other *L. rhamnosus* strains at genomic and phenotypic level. The strain GR-1 was originally isolated from a female urethra, and was assessed with *L. rhamnosus* GG from a feces sample of a healthy male, and *L. rhamnosus* LC705 from a dairy product. A key difference is the absence in GR-1 and LC705 of the *spaCBA* locus required for pili-mediated intestinal epithelial adhesion. In addition, the *L. rhamnosus* GR-1 genome contains a unique cluster for exopolysaccharide production, which is postulated to synthesize glucose-rich, rhamnose-lacking exopolysaccharide molecules that are different from the galactose-rich extracellular polysaccharide of *L. rhamnosus* GG. Compared to *L. rhamnosus* GG, *L. rhamnosus* GR-1 was also genetically predicted and experimentally shown to better metabolize lactose and maltose, and to better withstand oxidative stress, which is of relevance in the vagina. This study could thus provide a molecular framework for the selection of the optimal probiotic strain for each targeted niche and condition, but further substantiation of niche adaptation mechanisms of lactobacilli is warranted.

## Introduction

The importance of *Lactobacillus* species in promoting a healthy ecosystem in the human vagina has been well-recognized (Bruce et al., 1973; Petrova et al., 2015). The ability to replenish an aberrant vaginal microbiota with exogenous lactobacilli as probiotics was first reported in 1988 using *Lactobacillus rhamnosus* GR-1 (Bruce and Reid, 1988). The organism had been isolated from the distal urethra of a healthy female, and shown to counter the growth and adhesion to uroepithelial cells of urinary pathogens, and reduce urinary infection in animas (Chan et al., 1985; Reid et al., 1985). Since then, other attributes of the strain that appear to be important in counteracting pathogens have been reported, such as penetration of pathogenic biofilms (McMillan et al., 2011) via, for example, lectin-like proteins (Petrova et al., 2016), and enhancement of host defenses (Kirjavainen et al., 2008). The strain has also been shown to temporarily colonize the human vagina (Gardiner et al., 2002) and intestine *in vivo* following oral uptake (Reid et al., 2003). The fact that oral application of *L. rhamnosus* GR-1 can result in vaginal colonization is of interest in view of the natural ascension of lactobacilli from the gastro-intestinal to the vaginal tract.

As our understanding of lactobacilli, probiotics and the vaginal microbiota has evolved, it is becoming possible to explore the mechanisms that are key for successful applications to humans. Therefore, in the present study, we report the genome sequence of *L. rhamnosus* GR-1 and compare it to that of the gastro-intestinal probiotic *L. rhamnosus* strain GG (Kankainen et al., 2009), the model dairy isolate *L. rhamnosus* LC705 (Kankainen et al., 2009) as well as the other related *L. rhamnosus* strains currently in the NCBI database. This was done for two reasons: to investigate (i) inter-strain differences, and (ii) why *L. rhamnosus* GR-1 appears to be better adapted to the vagina, but less to the intestinal tract, than *L. rhamnosus* GG (Gardiner et al., 2002; Colodner et al., 2003). Insight into the genome of *L. rhamnosus* GR-1, and its comparison with related strains from other isolation sources, may help the identification of molecules playing roles in host–microbe and microbe–microbe interactions and the elucidation of the strain’s functional and niche-adaptation mechanisms. This could particularly be of interest for approaches that aim at evading pathogens in the vaginal niche, as exemplified in our recent manuscript (Petrova et al., 2016). Furthermore, detailed knowledge about the genome of *L. rhamnosus* GR-1 will provide a better understanding on the colonization and adaptation capacity of this strain following probiotic administration.

## Materials and Methods

### Bacterial Strains and Growth Conditions Used in This Study

Lactobacilli were grown standard at 37°C in MRS medium (Difco) in non-shaking conditions. Modified de Man Rogosa Sharpe medium (mMRS), in which glucose was replaced by the same concentration of varied sugars (20 g/L), was used as a growth medium to determine differences in the sugar utilization between *L. rhamnosus* GG, *L. rhamnosus* GR-1, and *L. rhamnosus* LC705. Each sugar compound was tested in three biological repetitions and each of the experiment was repeated at least three times.

### DNA Isolation

*Lactobacillus rhamnosus* GR-1 was cultured in MRS agar (Sigma-Aldrich, Belgium), incubated at 37°C micro-aerophically for 24 h, and DNA prepared using Epicentre MasterPure^TM^ DNA Purification Kit and dsDNA quality checked with eppendorf UV Biophotometer (Oceanside, CA, United States).

### Sequencing and Annotation

Purified DNA was sent to Illumina (Illumina, Inc., San Diego, CA, United States) for library preparation and sequencing. We received 35 bp paired-end reads which were used for assembly by Velvet 1.0.13 using a kmer of 27, expected coverage of 40, and a coverage cutoff of 20. Using the draft assembly, open reading frames (ORFs) were first predicted and then annotated with GeneMark v.4.21 and additionally predicted by Glimmer. The genomic assembly is available under the ENA Accession No. PRJEB24764.

### Pan-Genome Analysis

All available *L. rhamnosus* genomic assemblies (NCBI; 04/04/2017) were downloaded and their quality was evaluated using QUAST v4.3 (Gurevich et al., 2013). This resulted in 116 different genomic assemblies that were downloaded, of which 100 + 1 (*L. rhamnosus* GR-1) passed quality control. These analyses included visualization of different quality control parameters (such as GC content, total genome length, N50 and number of N’s per 100.000 bases). Only genomes with a N50 > 10 kb and a number of N’s per 100.000 bases lower than 500 were kept. In addition, the completeness of all genomes was checked using CheckM (Parks et al., 2015) and only genomes with a completeness >95% were kept. The assemblies were annotated using Prokka v1.12 (Seemann, 2014) and the pan genome was calculated with Orthofinder v1.1.18 (Emms and Kelly, 2015) using the -M msa option. For further analysis, a core orthogroup is defined as an orthogroup present in >95% of the genomes. The phylogenetic tree was constructed with RAxML v8.2.9 (Stamatakis, 2014) using the alignment of all single copy core orthogroups on amino acid level, after addition of *Lactobacillus casei* subsp. *casei* ATCC 393 (GCA_000829055) as outgroup, PROTCATWAG as substitution matrix and the autoMRE option. The gene content was further analyzed in R and visualized using the R package UpsetR v1.3.3. Furthermore, the functional capacity was explored by mapping all representative sequences against the eggNOG (v4.5) *Bacillus* database (Huerta-Cepas et al., 2016) and calculating distance matrices using the R Package vegan v2.4.4 as described in Wuyts et al. (2017). Hereby, representative sequences represent the most abundant sequence in each orthogroup. Visualization was done using the R package ggplot2 v2.2.1 (Wickham, 2009).

### Adhesion Assay to CaCo-2 and VK2/E6E7 Cells

*In vitro* adhesion assays using the CaCo-2 (ATCC HTB 37^TM^) and VK2/E6E7 (ATCC CRL-2616^TM^) cell line were performed as previously described (Petrova et al., 2016). Briefly, differentiated Caco2 or VK2/E6E7 cells were washed two times with pre-warmed 1x PBS and subsequently incubated for 1 h with 1.5 ml of 10^7^ bacterial cells. After incubation at 37°C for 1 h, the epithelial cell cultures were washed twice with pre-warmed PBS. Subsequently, 100 μl of trypsin-EDTA (1x) (Invitrogen) was added to each well and incubated for 10 min at 37°C. Finally, 900 μl of PBS was added, mixed and serial dilutions were plated out. Plates were incubated at 37°C for 72 h. The adhesion ratio (percentage) was calculated by comparing the number of adherent cells to the cell number of the added original bacterial suspension (10^7^ CFU/ml). Adhesion of *L. rhamnosus* GG, *L. rhamnosus* GR-1 and *L. rhamnosus* LC705 was tested in triplicate in three independent experiments.

### *spaCBA* Presence

The presence of *spaA* (NCBI Protein database Accession No. BAI40953), *spaB* (BAI40954) and *spaC* (BAI40955) genes was evaluated by performing a BLASTp (Camacho et al., 2009) search against a database containing all protein sequences of the above mentioned genomic assemblies. Only hits with a percentage identity and query coverage higher than 80% were retained.

### EPS Isolation and Monomer Characterization

Extracellular polysaccharide (EPS) molecules were isolated and quantified as described previously (Lebeer et al., 2009). Briefly, total EPS was extracted from *L. rhamnosus* GG and *L. rhamnosus* GR-1 cells by mild sonication followed by ethanol precipitation and dialysis against water [6,000- to 8,000-Da dialysis membrane (Spectra/Por, VWR International)]. The total amount of carbohydrate was estimated by the phenolsulfuric acid method. The neutral sugar monomer composition of the isolated polysaccharides was determined according to the method of Englyst and Cummings (1984) by gas chromatography after hydrolysis and derivatization to alditol acetates. β-D-Allose was used as an internal standard and calibration samples (glucose, galactose, rhamnose, and mannose) containing the expected monosaccharides were included with each set of samples.

### Induction of Cytokine Gene Expression in VK2/E6E7 Epithelial Cells

The cytokine expression was performed as previously described (Lebeer et al., 2012) Briefly, VK2/E6E7 cells growing in 12-well tissue culture plates were deprived of FBS 1 day before the mRNA induction experiments. *L. rhamnosus* GG, *L. rhamnosus* GR-1, and *L. rhamnosus* LC705 cells were grown overnight in MRS medium and subsequently centrifuged at 2,000 × *g* at 4°C for 10 min. After washing with 1x PBS, cells were resuspended in DMEM without FBS and adjusted to a final concentration of 1 × 10^7^ CFU/ml. A 1.5-ml volume of the *L. rhamnosus* GG or *L. rhamnosus* GR1 cell suspension was then added to the VK2/E6E7 epithelial cells for 1.5 h. Afterward, the epithelial cells were rinsed twice with pre-warmed 1x PBS and subsequently 0.2-ml volume of PBS was added to each of the wells. RNA was extracted from the VK2/E6E7 cells by using the high pure RNA isolation kit (Roche) following the manufacturer’s protocol. The cytokine genes expression were measurement by quantitative reverse transcription-PCR (qRT-PCR) by using reverse transcription (SuperScript III first-strand synthesis system; Invitrogen), and real-time DNA amplification (TaqMan universal PCR master mix; Applied Biosystems). For RT-qPCR amplification, the StepOnePlus Real Time PCR system (Applied Biosystems, Lennik, Belgium) was used. All primers and probes were designed based on published sequences and chemically synthesized by Integrated DNA Technologies (IDT) (Belgium) (**Table 1**). Purified plasmid DNA specific for each targeted cytokine gene served as cDNA plasmid standards and was used to quantify the respective cytokine in the test samples. Peptidylprolyl *cis–trans* isomerase A (PPIA), served as the housekeeping gene.

**Table 1:** Primers and probes used in this study for cytokine mRNA measurements.

Target mRNA	Sequence (5′–3′)	Primer or probe	Reference
PPIA	CGCGTCTCCTTTGAGCTGTT	FW^∗^	Hayeshi et al., 2008
	CTGACACATAAACCCTGGAAT AATTC	RV	
	CAGACAAGGTCCCAAAGACAGCAGAAAATTT	TaqMan probe	
IL-8	TGGCAGCCTTCCTGATTTCT	FW	Bullens et al., 2006
	TTAGCACTCCTTGGCAAAACTG	RV	
	CAGCTCTGTGTGAAGGT	TaqMan probe	
TNF	TCTTCTCGAACCCCGAGTGA	FW	Giulietti et al., 2001
	CCTCTGATGGCACCACCAG	RV	
	TAGCCCATGTTGTAGCAAACCCTCAAGCT	TaqMan probe	
IL-6	GAGGATACCACTCCCAACAGACC	FW	Bullens et al., 2006
	AAGTGCATCATCGTTGTTCATACA	RV	
	CAGAATTGCCATTGCACAACTCTTTTCTCA	TaqMan probe	

^∗^*FW, forward primer; RV, reverse primer; TaqMan probe, dually labeled with 5′ 6-carboxyfluorescein and 3′ 6-carboxytetramethylrhodamine; PPIA, peptidyl-prolyl *cis–trans* isomerase A; IL-8, interleukin-8; TNF, tumor necrosis factor*.

### Stress Survival Assays in Simulated Gastro-Intestinal and Vaginal Conditions

Stress survival tests were performed as previously described (Lebeer et al., 2011). Briefly, simulated gastric juice was prepared using 3.5 g/l glucose, 2.05 g/l NaCl, 0.60 g/l KH_2_PO_4_, 0.11 g/l CaCl_2_, and 0.37 g/l KCl, adjusted to pH 2.0 using 1.0 M HCl, and autoclaved at 121°C for 15 min (Corcoran et al., 2005). Subsequently, 0.05 g/l porcine bile (Sigma-Aldrich), 0.1 g/l lysozyme (Sigma-Aldrich), and 13.3 mg/l pepsin (Sigma-Aldrich) were added as stock solutions prior to analysis.

For the stress survival assays, 10^8^ CFU/ml (based on estimations via optical density at 600 nm) cells were resuspended in the appropriate volume of either simulated gastric juice or 0.1% vol/vol H_2_O_2_. Suspensions were incubated at 37°C for 90 min with constant stirring. The percentage of survivals was calculated by comparing the exact colony forming units (plate counts) before and after addition to simulated gastric juice or 0.1% H_2_O_2_. Phosphate-buffered saline (PBS) at pH 7.4 was used as baseline (negative control). Each strain and/or condition was tested threefold, and each experiment was performed at least in triplicate.

## Results

### Phylogenetic Relationships Between *L. rhamnosus* Strains

The draft genome sequence of *L. rhamnosus* GR-1 was determined based on the assembly of 266 contigs, and found to have a genome size of 2.89 Mbp, a whole genome GC % of 46.48% and a total of 2,714 predicted genes. The pan genome of *L. rhamnosus* was calculated using the *L. rhamnosus* GR-1 assembly and 100 publicly available strains [10 complete genomes and 90 + 1 (LGR1) draft genomes]. Only genomes with a sufficient high completeness were kept for the analysis to avoid problems with the absence of certain genes. In total, this pan genome contained 281,225 genes which were clustered into 4,526 orthogroups, defined as the group of genes descended from a single gene in the most recent common ancestor (Emms and Kelly, 2015). 2,143 orthogroups were found to be core orthogroup, while the remaining 2,383 were identified as accessory.

**FIGURE 1:**
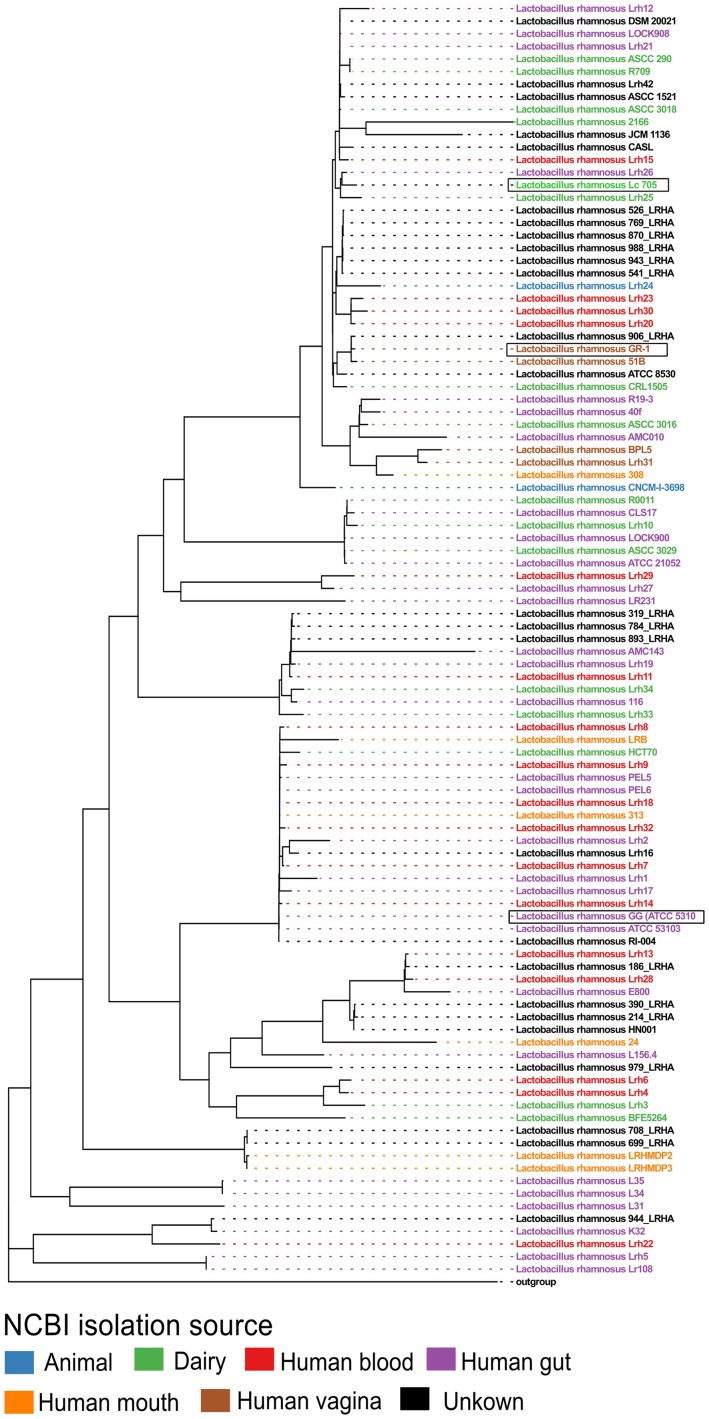
Phylogenetic tree of all publicly available *Lactobacillus rhamnosus* strains including *L. rhamnosus* GR-1, based on the amino acid sequences of 952 single copy core orthogroups with *Lactobacillus casei* subsp. *casei* ATCC 393 (GCA_000829055) as outgroup. The branch length of the outgroup was shortened for visualization purposes.

Next, all single-copy core orthogroups (952 in total) were used to construct a high resolution phylogenetic tree (**Figure 1**). *Lactobacillus casei* subsp. *casei* ATCC 393 (GCA_000829055) was chosen as outgroup, based on the most recent comparative genomics study (Wuyts et al., 2017). As shown in **Figure 1**, strain *L. rhamnosus* GR-1 shows high similarity with *L. rhamnosus* strain 51B (isolated from the vagina of a healthy Russian woman) and strain *L. rhamnosus* 906_LRHA (origin unclear) based on their 952 single copy core orthogroups. Similarly, strains *L. rhamnosus* Lc705, *L. rhamnosus* Lrh25, and *L. rhamnosus* Lrh26 are phylogenetically very closely related, as well as *L. rhamnosus* GG (ATCC 53103) and 17 other *L. rhamnosus* strains. Of note, in addition to *L. rhamnosus* GR-1 and *L. rhamnosus* 51B, two other genomic assemblies were annotated as vaginal isolates (strains BPL5 and Lhr31), which also seem to cluster together in a separate small clade. In contrast, isolates from all other isolation sources seem to be spread all over the phylogenetic tree.

### Phylogenetic Relationships Between Three Well-Known and Widely Used *L. rhamnosus* Strains

**FIGURE 2:**
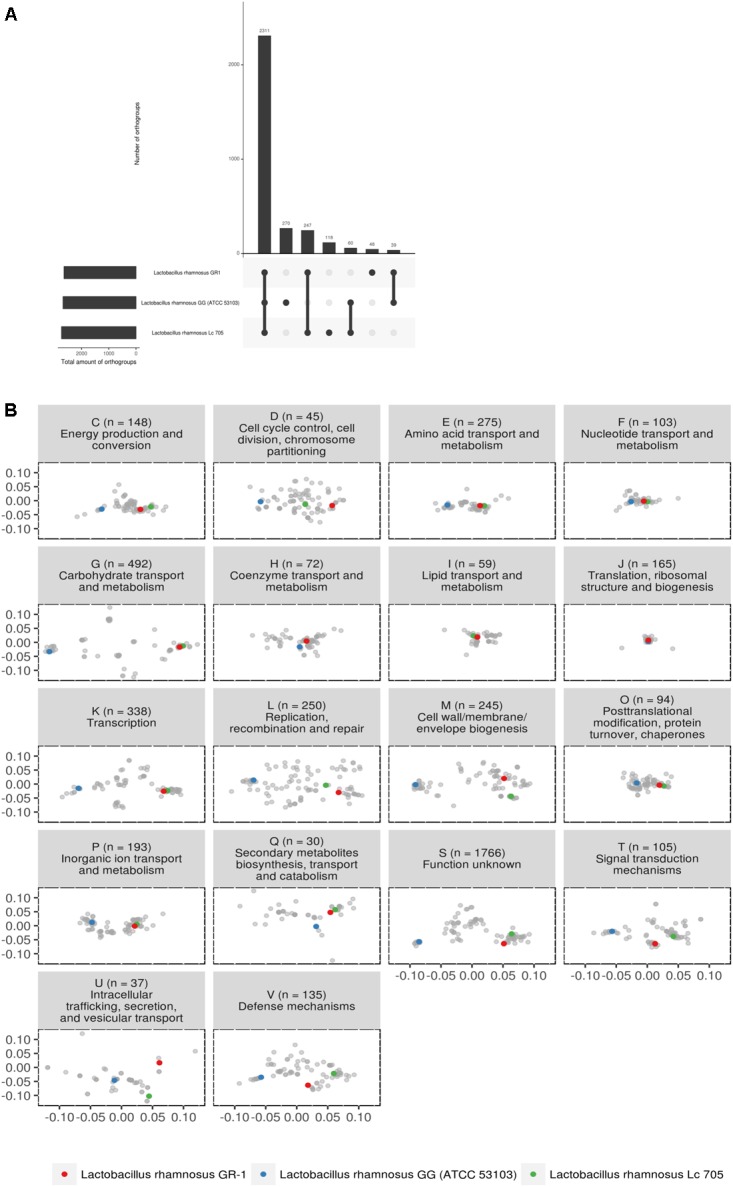
**(A)** Upset plot comparing all shared orthogroups of the three studied probiotic strains. **(B)** PCoA of predicted functional capacity of all *L. rhamnosus* strains based on mapping of all orthogroups to the eggNOG database (v4.5) (Huerta-Cepas et al., 2016). Each letter represents a different functional category, as defined above each plot together with the amount of orthogroups used for that functional category. Some orthogroups mapped to multiple functional categories. The majority of the orthogroups (1,766 of them) mapped to category S (function unknown).

Subsequently, we focused in detail on the phylogenetic relationship between strains GR-1, GG and LC705, as these are widely studied and commercially available probiotics with different isolation source. The three strains appear to have 2,311 orthogroups in common (**Figure 2A**). *L. rhamnosus* GR-1 is missing 60 orthogroups between *L. rhamnosus* GG and *L. rhamnosus* LC705. Strains *L. rhamnosus* LC705 and *L. rhamnosus* GR-1 have 247 orthogroups in common which are not present in *L. rhamnosus* GG. Within this latter group, many metabolic genes related to carbohydrate metabolism and complex protein degradation are present. Strains *L. rhamnosus* GG and *L. rhamnosus* GR-1 have 39 orthogroups in common which are not present in *L. rhamnosus* LC705, suggesting that they are putative factors of relevance within the human body. Of most importance to our objectives, *L. rhamnosus* GR-1 has 48 unique orthogroups not present in *L. rhamnosus* GG or *L. rhamnosus* L705, while *L. rhamnosus* GG has 270 unique orthogroups not present in *L. rhamnosus* GR-1 or LC705.

The predicted functional capacity of the three probiotic strains was further studied in relationship to all other *L. rhamnosus* genomes by mapping all orthogroups to the eggNOG database (v4.5) (Huerta-Cepas et al., 2016). **Figure 2B** shows that for most functional categories, the genomes clustered according to the phylogeny described in **Figure 1**. In general, the distance between strain GR-1 and LC705 is much smaller than the distance between GR-1 and strain GG. However, some categories show overlapping orthogroup composition, pointing toward functional conservation between *L. rhamnosus* GG, *L. rhamnosus* GR-1, and *L. rhamnosus* LC705. These include category F, representing nucleotide transport and metabolism; category H covering coenzyme transport and metabolism; category I representing lipid transport and metabolism; category J, covering translation, ribosomal structure and biogenesis function; and category O, representing post-translational modification and chaperons. On the other hand, and of special interest, are the functional categories that show multiple clusters, suggesting a different functional capacity between these *L. rhamnosus* strains. For example, category K show that strain GR-1 and LC705 have a similar composition of orthogroups related to transcription, while strain GG seems to show a different composition. Likewise category G, representing orthogroups related to carbohydrate transport and metabolism, suggests a different carbohydrate usage between the vaginal and dairy strains (GR-1 and LC705) and strain GG.

### Carbohydrate Utilization

**FIGURE 3:**
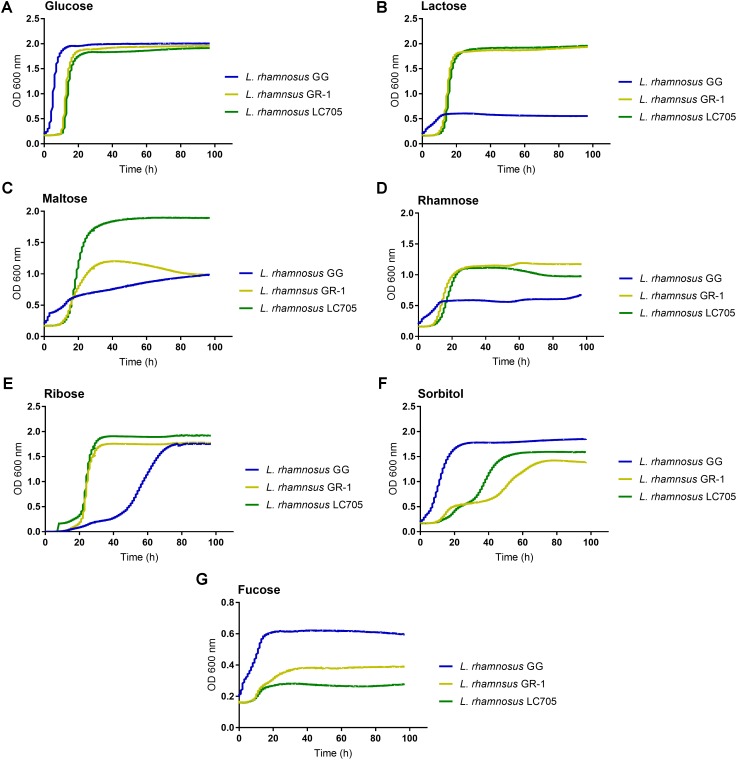
Comparison of the metabolic capacity of *L. rhamnosus* GG, *L. rhamnosus* GR-1, and *L. rhamnosus* LC705. All the experiments were performed in modified MRS in which the main carbon source being glucose **(A)** was replaced with lactose **(B)**, maltose **(C)**, rhamnose **(D)**, ribose **(E)**, sorbitol **(F)**, and fucose **(G)**.

Since large differences in carbohydrate metabolism-encoding genes were found between *L. rhamnosus* GG and *L. rhamnosus* GR-1, with the latter having most of its carbohydrate utilization capacity in common with the dairy strain *L. rhamnosus* LC705, we subsequently experimentally validated major differences in carbohydrate utilization between these strains (**Figure 3A**).

In contrast to *L. rhamnosus* GG, the *L. rhamnosus* GR-1 genome contains unique genes for lactose utilization, similar to *L. rhamnosus* LC705, and we could confirm experimentally that *L. rhamnosus* GR-1 can ferment lactose while *L. rhamnosus* GG cannot (**Figure 3B**). *L. rhamnosus* GR-1 appears to also more efficiently metabolize maltose (**Figure 3C**). In addition, *L. rhamnosus* GR-1 is able to metabolize rhamnose (**Figure 3D**). *L. rhamnosus* GR-1 also showed to have an enhanced capacity to metabolize ribose in agreement with LGR-1 having genes *GR1_01559–GR1_01561* (**Figure 3E**). Furthermore, *L. rhamnosus* GR-1 shows a typical bi-phase growth curve when sorbitol is provided as main sugar source (**Figure 3F**). *L. rhamnosus* GR-1, similar to *L. rhamnosus* LC705, was not able to grow on fucose, in comparison to *L. rhamnosus* GG which shows a slow but significant growth in the presence of this carbon source (**Figure 3G**). Finally, the growth of *L. rhamnosus* GR-1 and *L. rhamnosus* GG was examined in the presence of glycogen, which was previously reported to be an important sugar source in the vaginal niche. However, no growth was observed for both of the strains under the tested condition (data not shown).

### Adhesion and Presence of SpaCBA Pili

**FIGURE 4:**
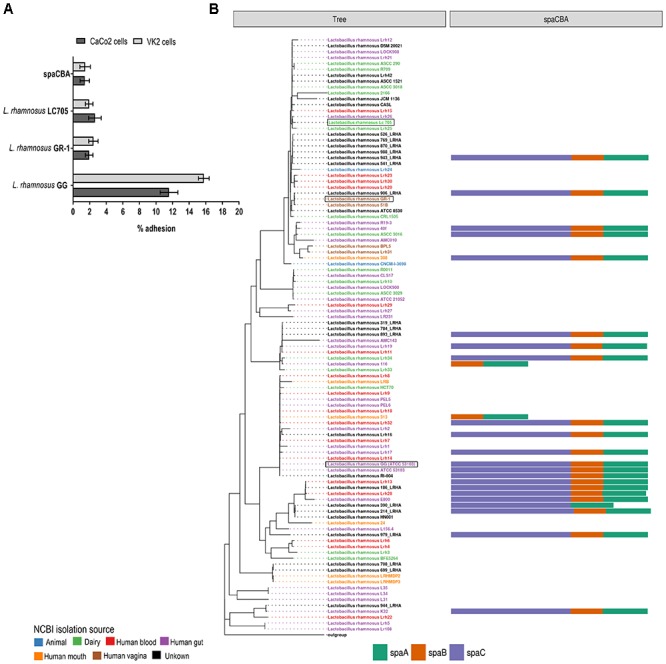
**(A)** Adhesion to intestinal CaCo2 cells and vaginal VK2 epithelial cells of *L. rhamnosus* GG, *L. rhamnosus* GR-1 and *L. rhamnosus* LC705. **(B)** Presence of SpaCBA pili-encoding genes in all publicly available *L. rhamnosus* strains.

The *spaCBA*-encoded pili that are the key adhesins for *L. rhamnosus* GG to bind to human intestinal mucus and intestinal epithelial cells (Kankainen et al., 2009; Lebeer et al., 2012), are absent from the *L. rhamnosus* GR-1 and *L. rhamnosus* LC705 genome sequence, as confirmed by PCR. This is also reflected by the phenotypic comparison, in which *L. rhamnosus* GR-1 and *L. rhamnosus* LC705 behave like the *L. rhamnosus* GG *spaCBA* gene deletion mutant lacking pili in terms of adhesion to intestinal epithelial CaCo-2 cells and VK/E6E7 vaginal epithelial cells (**Figure 4A**). In addition, mining other publicly available genome sequences of vaginal *L. rhamnosus* strains suggest that they all lack pili (**Figure 4B**). Of note, the presence of *spaCBA* genes is more prevalent in gastro-intestinal isolates than in dairy isolates. However, not all gastro-intestinal strains bear these *spaCBA* genes, confirming that pili form a strain-specific characteristic acquired during evolution.

### Extracellular Polysaccharide (EPS) Production and Immunomodulation

**FIGURE 5:**
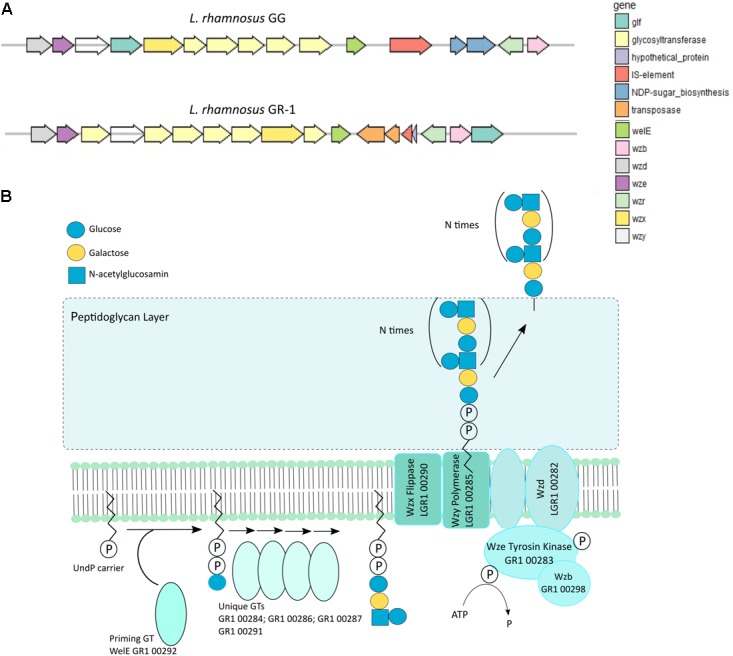
**(A)** Organization of the EPS gene cluster of *L. rhamnosus* GR-1 in comparison with the EPS gene cluster organization of *L. rhamnosus* GG. **(B)** Schematic representation of the putative steps in EPS biosynthesis by *L. rhamnosus* GR-1 that are encoded within the EPS gene cluster.

**FIGURE 6:**
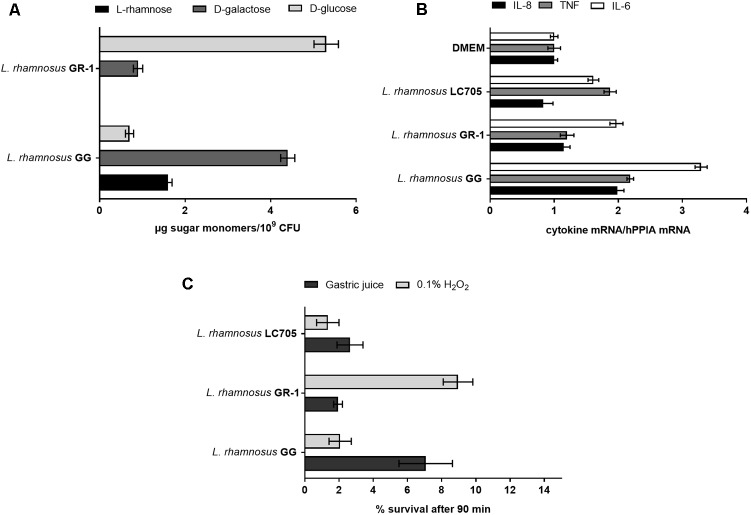
Phenotypic comparison of *L. rhamnosus* GG, *L. rhamnosus* GR-1, and *L. rhamnosus* LC705. **(A)** EPS monomer composition analysis of *L. rhamnosus* GG versus *L. rhamnosus* GR-1. **(B)** IL-8, TNF and IL-6 mRNA induction in VK2/E6E7 epithelial cells after incubation for 1 h with *L. rhamnosus* GG, *L. rhamnosus* GR-1, and *L. rhamnosus* LC705. **(C)** Stress resistance of *L. rhamnosus* GG, *L. rhamnosus* GR-1 and *L. rhamnosus* LC705 in simulated gastric juice and 0.1% H_2_O_2_, represented as survival after 90 min incubation.

Extracellular polysaccharide production is an important adaptation factor in lactobacilli. The galactose-rich rhamnose-containing EPS molecules of *L. rhamnosus* GG form a protective shield against innate immune factors in the intestine including cathelicidins and complement factors (Lebeer et al., 2011). Therefore, a EPS cluster comparative analysis was performed for *L. rhamnosus* GG versus *L. rhamnosus* GR-1 (**Figure 5A**). Similarity can be observed for genes predicted to encode for the regulation of EPS production and polymerization, i.e., genes *wzd* and *wze* located in the beginning of the EPS operon and *wzr* and *wzb* genes located in the end of the cluster. The EPS cluster of *L. rhamnosus* GR-1 appears to further contain unique glycosyltransferases for the steps of EPS biosynthesis (schematically represented on **Figure 5B**). The major differences between the two strains was the absence of NDP-sugar biosynthesis genes in the genome of *L. rhamnosus* GR-1 (represented with blue arrows in **Figure 5A**). The NDP-sugar biosynthesis genes were predicted to be involved in the synthesis of galactose-rich rhamnose- containing EPS cluster in *L. rhamnosus* GG (Lebeer et al., 2009). To experimentally compare the neutral sugar monomer composition, cell bound EPS was isolated from *L. rhamnosus* GR-1 and *L. rhamnosus* GG. In contrast to the galactose-rich rhamnose-containing EPS molecules of *L. rhamnosus* GG (Lebeer et al., 2009), those of *L. rhamnosus* GR-1 are glucose-rich and completely lack rhamnose (**Figure 6A**), in agreement with the absence of *rml*A-D genes from its genome sequence (**Figure 5A**). We were able to experimentally show that *L. rhamnosus* GR-1 mediates different cytokine responses in vaginal VK2/E6E7 epithelial cells compared to *L. rhamnosus* GG (**Figure 6B**). While *L. rhamnosus* GG and *L. rhamnosus* LC705 induced the mRNA expression of the pro-inflammatory markers tumor necrosis factor (TNF) mRNA (twofold above background), *L. rhamnosus* GR-1 did not under the tested conditions. Further *L. rhamnosus* GG, but not *L. rhamnosus* LC705 and *L. rhamnosus* GR-1, was able to induce interleukin-8 (IL-8) with twofold above background. All tested strain induced IL-6 with twofold above background for *L. rhamnosus* LC705 and *L. rhamnosus* GR-1 and 3.5-fold for *L. rhamnosus* GG (**Figure 6B**).

### Stress Resistance in Simulated Gut and Vaginal Conditions

Depending on the probiotic administration method, surviving gastric acidity is a key determinant for optimal survival for *L. rhamnosus* GG to reach the intestine and *L. rhamnosus* GR-1, taken orally to reach the rectum and vagina. In simulated gastric juice, *L. rhamnosus* GG was shown to survive significantly better than *L. rhamnosus* GR-1 and *L. rhamnosus* LC705 (**Figure 6C**). On the other hand, *L. rhamnosus* GR-1 showed a better survival capacity under oxidative stress, particularly relevant in the vagina, as experimentally documented by an significantly enhanced resistance to 0.1% vol/vol hydrogen peroxide (H_2_O_2_) compared to *L. rhamnosus* GG and *L. rhamnosus* LC705 (**Figure 6C**). This phenotype might be related to the presence of a putative thioredoxin genes (*GR1_00460, GR1_01468*, and *GR1_01733*) in *L. rhamnosus* GR-1.

## Discussion

While the genome sequences of various gastro-intestinal and dairy probiotic *Lactobacillus* strains have been published since 2004, including *Lactobacillus acidophilus* NCFM (Altermann et al., 2005), *Lactobacillus salivarius* subsp. *salivarius* UCC118 (Claesson et al., 2006) *Lactobacillus gasseri* ATCC 33323 (Azcarate-Peril et al., 2008) and *L. rhamnosus* GG (Kankainen et al., 2009), the urogenital probiotic strains are lagging behind. Nevertheless, probiotic application of lactobacilli hold greater promise in the female vagina, because of the dominance of lactobacilli in these niches under health conditions, and their depletion upon disease (Petrova et al., 2015).

Here we report that *L. rhamnosus* GR-1 has a genome size of 2.91 Mbp, in agreement with other *L. rhamnosus* strains. For comparison, *L. iners* has only a genome size of only 1.3 Mbp (Macklaim et al., 2011) reflecting large reductive genome evolution (Frese et al., 2011) and a potential symbiotic lifestyle (Petrova et al., 2017). Even though we only know the source of isolation of these strains, the present genome comparison of *L. rhamnosus* GR-1, *L. rhamnosus* GG, and *L. rhamnosus* LC705 and the other *L. rhamnosus* genomes publicly available gives a snapshot of which predicted functional capacity correlates with the preferred niche of these strains.

Most apparent is the presence of pili-encoding genes for intestinal mucus adhesion only in *L. rhamnosus* GG, and not in *L. rhamnosus* GR-1 and *L. rhamnosus* LC705, in agreement with the fact that the energy-expensive biosynthesis of these long heteropolymeric structures only provides a competitive advantage in the gastro-intestinal tract. Perhaps the absence of pili and relatively low adhesion capacity of *L. rhamnosus* GR-1 compared to *L. rhamnosus* GG might actually promote the natural ascension of this strain from the gastro-intestinal tract to the vagina via the rectum, since it was previously shown *in vivo* that *L. rhamnosus* GG is not able to colonize the human vagina as efficiently as *L. rhamnosus* GR-1 (Gardiner et al., 2002; Colodner et al., 2003). Our results are in agreement with the previous comparative genomic analysis of *L. rhamnosus* strains in which the *spaCBA* pili gene cluster was prevalent in human isolates, but rarely detected in dairy isolates (Douillard et al., 2013). Similarly, the authors observed that intestinal *L. rhamnosus* isolates were the most prevalent group encoding SpaCBA pili, but none of the vaginal and oral isolates produced pili (Douillard et al., 2013).

On the other hand, the *L. rhamnosus* GR-1 genome seems to encode a number of traits suited to vaginal persistence, unlike the gut targeted probiotic *L. rhamnosus* GG. Of note, the genome and functional analysis of *L. rhamnosus* GR-1 reflects a more versatile carbohydrate utilization capacity than *L. rhamnosus* GG. Strain *L. rhamnosus* GR-1 was indeed predicted and experimentally shown here to utilize maltose, lactose, ribose and rhamnose, carbohydrates, which were previously shown to be expressed by human vaginal tissue (Gregoire, 1963; Wang et al., 2015) and can therefore be used an important carbon source for vaginal species. Interestingly, these carbohydrates are also highly relevant for dairy applications and were preferable carbon source for *L. rhamnosus* LC705. In contrast, we observed that *L. rhamnosus* GG utilize fucose better compared to *L. rhamnosus* GR-1 and *L. rhamnosus* LC705, as also reported by other authors (Douillard et al., 2013). This highlights the importance of fucose as carbon source in the intestinal tract, where fucosylated compounds such as human mucin and other glycoproteins are highly abundant.

In addition, we were able to show that *L. rhamnosus* GR-1 can withstand much better oxidative stress in comparison with *L. rhamnosus* GG. This might be important for better survival and adaptation in the vaginal niche. Notably, *L. rhamnosus* GG survived better in simulated gastric juice than *L. rhamnosus* GR-1. This low survival capacity of *L. rhamnosus* GR-1 might affect its capacity to reach the intestine, rectum, and vagina to help prevent or treat vaginally associated disorders, such as bacterial vaginosis, aerobic vaginitis and yeast infections. Therefore, applying *L. rhamnosus* GR-1 vaginally, as capsules, might be a better choice for the treatment of vaginal disorders and infections. This will result in a local administration of the strain and will ensure its better survival and colonization capacity, probably also resulting in a better treatment outcome. Yet, orally administrated tablets or capsules are better accepted by patients and still show an improvement of vaginal diseases. Hereto, they are often given twice daily at concentration of at least 10^9^ CFU, which may guarantee the survival and the passage of sufficient numbers of *L. rhamnosus* GR-1 to the vaginal niche. Of note, vaginally administrated capsules are often applied only once a day which may also lower the cost of the treatment. Nevertheless, future studies should focus on the effect of orally vs. vaginally applied *L. rhamnosus* GR-1 to confirm which administration route is more effective.

Another striking difference is that the *L. rhamnosus* GR-1 genome encodes at least one EPS-associated cluster, and was shown to produce glucose-rich EPS molecules that lack rhamnose. The EPS gene cluster of *L. rhamnosus* GG (*LGG_02036–LGG_02054*), encoding galactose-rich EPS molecules, is important in survival in the gastro-intestinal tract, by protecting against innate immune factors (Lebeer et al., 2011). Our present finding suggests there could be different recognition of the two *L. rhamnosus* strains by immune cells in the gut and in the vagina. The Type I mucosal surface in the gut has a simple columnar epithelium, while the type II mucosal surface in the vagina and ectocervix have a protective squamous epithelial layer lacking IgA transport mechanisms. In addition, while the submucosa of the Type I surface is rich in mucosa-associated lymphoid tissue, the Type II mucosal surface has specialized highly phagocytic Langerhans cells in the epithelium, yet only contains a sparse network of dendritic cells, macrophages and rare lymphocytes in the submucosa (Iwasaki, 2010). We showed here that *L. rhamnosus* GR-1 did not induce expression of TNF and IL-8 by VK2/E6E7, while *L. rhamnosus* GG was able to induce expression of TNF, IL-8 and IL-6 under the tested conditions, indicating a different recognition of the two strains by epithelial cells.

Interesting, Douillard et al. (2013) reported that *L. rhamnosus* species can be separated in two main geno-phenotypes called A and B, which correspond to adaptation to different niches. The strains belonging to the geno-phenotype A were characterized by a lack of SpaCBA pili and the capacity to utilize lactose, maltose, and rhamnose. According to our results, *L. rhamnosus* GR-1 can be classified within A geno-phenotype. The authors observed that other vaginal and dairy strains can also be classified as A geno-phenotype or as strains adapted to more stable nutrient-rich environment, such as the vaginal niche. These strains appear to have lost various biological functions such as antimicrobial activity, stress resistance, adaptability and fitness to a range of habitats, all of which may not be required in the vaginal niche where lower bacterial diversity is observed. The geno-phenotype B, the one in which *L. rhamnosus* GG is classified, depicts strains adapted to the intestinal niche, characterized by production of pili, bile resistance and utilization L-fucose. These strains show adaptation to more diverse environments with constant changes in nutrition source, bacterial density, and host effects.

In summary, whilst *L. rhamnosus* is not a dominant species in the vagina of healthy women (Zhang et al., 2012), the selection of *L. rhamnosus* GR-1 as a probiotic, originally because of antagonism to uropathogens, is supported by the finding of a number of characteristics well-suited to vaginal persistence (Cadieux et al., 2002) and clinical effectiveness (Gardiner et al., 2002; Irvine et al., 2010). By identifying probiotic strain capabilities, it will 1 day be possible to improve the alignment of the best strain suited for a particular aberration in the vaginal niche.

## Author Contributions

MP, SL, and GR designed the experiments and wrote the manuscript. SW performed the genome comparison analysis and wrote part of the manuscript. JM, GG, and GR sequenced and annotated the genome of *L. rhamnosus* GR-1. MP and TV performed the experimental work. All the authors reviewed the manuscript and included additional suggestions and corrections.

## Conflict of Interest Statement

The authors declare that the research was conducted in the absence of any commercial or financial relationships that could be construed as a potential conflict of interest.
